# Transferring Plasmon Effect on a Biological System: Expression of Biological Polymers in Chronic Rejection and Inflammatory Rat Model

**DOI:** 10.3390/polym13111827

**Published:** 2021-05-31

**Authors:** Chien-Sung Tsai, Feng-Yen Lin, Yu-Chuan Liu, Yi-Wen Lin, Yi-Ting Tsai, Chun-Yao Huang, Shing-Jong Lin, Chi-Yuan Li, Cheng-Yen Lin, Horng-Ta Tseng, Chun-Min Shih

**Affiliations:** 1 Division of Cardiovascular Surgery, Tri-Service General Hospital, National Defense Medical Center, Taipei 115, Taiwan; sung1500@mail.ndmctsgh.edu.tw (C.-S.T.); cvsallen@mail.ndmctsgh.edu.tw (Y.-T.T.); 2 Department and Graduate Institute of Pharmacology, National Defense Medical Center, Taipei 115, Taiwan; 3 Taipei Heart Institute, Taipei Medical University, Taipei 110, Taiwan; g870905@tmu.edu.tw (F.-Y.L.); cyhuang@tmu.edu.tw (C.-Y.H.); sjlin888@tmu.edu.tw (S.-J.L.); b101107097@tmu.edu.tw (H.-T.T.); 4 Division of Cardiology and Cardiovascular Research Center, Taipei Medical University Hospital, Taipei 110, Taiwan; 5 Department of Internal Medicine, School of Medicine, College of Medicine, Taipei Medical University, Taipei 110, Taiwan; 6 Department of Biochemistry and Molecular Cell Biology, School of Medicine, College of Medicine, Taipei Medical University, Taipei 110, Taiwan; liuyc@tmu.edu.tw; 7 Cell Physiology and Molecular Image Research Center, Wan Fang Hospital, Taipei Medical University, Taipei 110, Taiwan; 8 Institute of Oral Biology, National Yang-Ming Chiao-Tung University, Hsinchu 711, Taiwan; ywlin@ym.edu.tw; 9 Department of Anesthesiology, China Medical University Hospital, Taichung 40447, Taiwan; cyli168@gmail.com; 10 Graduate Institute of Clinical Medical Science, China Medical University, Taichung 40402, Taiwan; 11 Healthcare Information and Management Department, Ming Chuan University, Taoyuan 333, Taiwan; a684094@ms28.hinet.net

**Keywords:** transferring plasmon effect, plasmon-activated water, orthotopic allograft transplantation, vasculopathy, endothelial cells

## Abstract

The plasmon-activated water (PAW) that reduces hydrogen bonds is made of deionized reverse osmosis water (ROW). However, compared with ROW, PAW has a significantly higher diffusion coefficient and electron transfer rate constant in electrochemical reactions. PAW has a boiling point of 97 °C and specific heat of0.94; the energy of PAW is also 1121 J/mol higher than ordinary water. The greater the force of hydrogen bonds between H_2_O, the larger the volume of the H_2_O cluster, and the easier it is to lose the original characteristics. The hydrogen bonding force of PAW is weak, so the volume of its cluster is small, and it exists in a state very close to a single H_2_O. PAW has a high permeability and diffusion rate, which can improve the needs of biological applications and meet the dependence of biological organisms on H_2_O when performing physiological functions. PAW can successfully remove free radicals, and efficiently reduce lipopolysaccharide (LPS)-induced monocytes to release nitric oxide. PAW can induce expression of the antioxidant gene Nrf2 in human gingival fibroblasts, lower amyloid burden in mice with Alzheimer’s disease, and decrease metastasis in mice grafted with Lewis lung carcinoma cells. Because the transferring plasmon effect may improve the abnormality of physiological activity in a biological system, we aimed to evaluate the influence of PAW on orthotopic allograft transplantation (OAT)-induced vasculopathy in this study. Here, we demonstrated that daily intake of PAW lowered the progression of vasculopathy in OAT-recipient ACI/NKyo rats by inhibiting collagen accumulation, proliferation of smooth muscle cells and fibroblasts, and T lymphocyte infiltration in the vessel wall. The results showed reduced T and B lymphocytes, plasma cells, and macrophage activation in the spleen of the OAT-recipient ACI/NKyo rats that were administered PAW. In contrast to the control group, the OAT-recipient ACI/NKyo rats that were administered PAW exhibited higher mobilization and levels of circulating endothelial progenitor cells associated with vessel repair. We use the transferring plasmon effect to adjust and maintain the biochemical properties of water, and to meet the biochemical demand of organisms. Therefore, this study highlights the therapeutic roles of PAW and provides more biomedical applicability for the transferring plasmon effect.

## 1. Introduction

Plasmon-activated water (PAW) is produced by allowing deionized reverse osmosis water (ROW) to flow through gold nanoparticles (AuNPs) under resonant illumination, which excites the hot electron transfer provided by AuNPs and destroys the hydrogen bonds between H_2_O [1,2]. PAW has a boiling point of 97 °C and specific heat of 0.94; the energy of PAW is also 1121 J/mol higher than ROW. The greater the force of hydrogen bonds between H_2_O, the larger the volume of the H_2_O cluster, and the easier it is to lose the original characteristics. Compared with ROW, PAW has higher physical and chemical activity, including greater solubility for alkali metal-chloride salts, increased ionic conductivity of NaCl, higher extraction efficiency of biological antioxidants, such as polyphenols, 2,3,5,4′-tetrahydroxystilbene-2-O-beta-d-glucoside (THSG), and polygonum multiflorum [3]. Intracellular ROS production and ROS-activated signaling are closely associated with the development of cancers, autoimmune diseases, and systemic inflammatory diseases. In recent years, scientists have found that PAW might reduce ROS-induced cellular and tissue damage mediated by various mechanisms [4]. In addition, PAW can inhibit the lipopolysaccharide (LPS)-induced production of nitric oxide (NO) by monocytic cells, thereby inhibiting oxidative stress during acute inflammatory responses [1]. It might also activate the Kelch-like ECH-associated protein 1 (Keap1)/nuclear factor erythroid 2 related factor 2 (Nrf2)/antioxidant response element (ARE) pathway and increase the antioxidant capacity of human gingival fibroblasts. Additionally, the administration of PAW may increase the survival rate of non-small-cell lung cancer (NSCLC)-bearing mice that are administered cisplatin, and reduce the metastasis of NSCLCs [5]. Furthermore, it might also reduce the myeloid burden in neurons and improve memory, as well as decrease the severity of Alzheimer’s disease in APPs we/PSEN1dE9 transgenic mice [6].

Orthotopic allograft transplantation (OAT) is the main treatment method for end-stage organ failure. Since the currently available clinical strategies can effectively control acute rejection, the short-term survival rate of patients after organ transplantation has greatly improved. However, presently, the main factor affecting the long-term survival of patients undergoing organ transplantation is chronic rejection. Chronic rejection is a process in which the alloimmune system attacks the donor graft, and causes continuous and diffused damage of the vessels, resulting in vasculopathy [7]. The targets of this alloimmune system attack include the epithelium and endothelium of the implanted graft. Arteries and microvessels, which cause the original normal parenchyma to be replaced by fibrotic scar, exhibit the hyperplasia of vascular intimal fibrosis that causes diffuse occlusion and narrowing of the vessels implanted in the graft [8]. Eventually, progressive arterial fibrosis and constriction will lead to organ ischemia, with the lesions leading to organ failure in patients after transplantation [9]. Therefore, OAT-induced vasculopathy is an important problem after organ transplantation that needs to be resolved.

The occurrence of chronic rejection after OAT is indeed related to the production of antibodies against donor-specific human leukocyte antigen (HLA) by the recipient [10]. Since OAT-induced vasculopathy only occurs in the vessels of the donor grafts that are transplanted, but not in the native vessels of the recipients, alloimmunity is currently considered to be the main cause of OAT-induced vasculopathy [8]. Donor antigen-presenting cells present in the donor organ, such as the MHC molecules on the surface of dendritic cells, will be recognized by the recipient’s T cells, which, in turn, results in the activation of the cellular immune response. In addition, the donor antigen fragments presented on the surface of the recipient’s antigen-presenting cells will be recognized by the recipient’s T cells [9]. This process is the main initiator of the immune response responsible for chronic rejection [11]. The activated T cells will release interleukin-2 (IL-2) and interferon-γ (IFN-γ). These cytokines will continue to activate the T cells, B cells, and macrophages, and additionally, the graft vascular endothelial cells will also be activated and will begin to express several vascular cell adhesion molecules. This process causes an increased number of recipient immune cells to be chemoattracted to the vascular endothelium of the donor graft, thereby amplifying the immune response [12]. The smooth muscle cells are also activated simultaneously, resulting in their proliferation [13] and the secretion and accumulation of extracellular matrix proteins, and concomitantly, the activated B cells produce anti-HLA antibodies, which combine with macrophages to jointly regulate the vascular damage of the implanted graft. Therefore, the damage after OAT, resulting from both T cell-associated cellular immune responses and B cell-associated humoral immune responses [11], eventually leads to vascular intimal hyperplasia, fibrosis [14], ischemia, and functional failure of the implanted graft [15].

Because PAW is safe, easily produced, and inexpensive, it should be a publicly accessible and feasible option. Based on our previous findings regarding the effectiveness of PAW in the treatment of inflammation-related diseases, we planned to further evaluate the effect of PAW on the occurrence of OAT-induced vasculopathy. In this study, we transplanted the thoracic aorta of PVG/Seac rats into the abdominal aorta of ACI/NKyo rats and compared the severity of vasculopathy after 30–150 days of OAT in animals that were administered ROW and PAW. We also compared the chronic rejection situation of animals in the two groups by immunohistochemical analysis of the spleen and explored the underlying mechanisms of OAT-induced vasculopathy. We expect that this study will enhance the applicability of PAW in the prevention and treatment of inflammatory diseases in the future.

## 2. Materials and Methods 

### 2.1. Preparation and Quality Examination of Plasmon-Activated Water

PAW was prepared as described previously [6,16]. Briefly, ROW (pH7.23, temperature 23.5 °C) was passed through a glass tube filled with AuNPs-adsorbed ceramic particles under illumination with green-light-emitting diodes (with wavelength maxima centered at 530 nm). Then, PAW was collected in glass sample bottles for subsequent use as soon as possible. To examine the purity of the prepared PAW, inductively coupled plasma-mass spectrometric (ICP-MS) analysis was performed, which indicated that the concentrations of the slightly dissolved metals in the PAW were ca. 0.62 ppb for Au, 43 ppb for Na, 25 ppb for K, 23 ppb for Al, 13 ppb for Mg, 4.5 ppb for Ca, and 0.41 ppb for Fe. Excluding Au, the total equivalent molar concentration of these dissolved metals was equal to ca. 6.9 × 10^−6^ N. This measured value was ca. 2.4 × 10^−7^ N for ROW as a reference. In addition, the slightly dissolved Au and the total equivalent molar concentrations of other dissolved metals in PAW (light-free) were 0.57 ppb and 5.2 × 10^−6^ N, respectively.

### 2.2. Animal Grouping and OAT Experiment

All animals were treated according to the protocols approved by the Institutional Animal Care Committee of the National Defense Medical Center (certificate no. IACUC-20-275). Experimental procedures and animal care conformed to the “Guide for the Care and Use of Laboratory Animals” published by the U.S. National Institutes of Health. PVG/Seac rat-to-ACI/NKyo rat OAT was performed as described previously [17] with some modifications, since this model displays OAT-induced vasculopathy similar to that observed in a clinical scenario [18] without acute vascular rejection as a major confounding factor. All animals were purchased from the National Bio Resource Project (NBRP), Kyoto, Japan. The animals used in this experiment were 8-week-old male PVG/Seac rats (NBRP rat no: 0080; donor rats) and ACI/NKyo rats (NBRP rat no: 0001; recipient rats) with 250–300 g body weight (BW). Rats were kept in microisolator cages on a 12-h day/night cycle and fed a normal rodent chow diet (scientific diet). Group 1 (ACI/NKyo sham control) consisted of ACI/NKyo rats that were fed a normal chow diet with ROW and were sham-operated, but did not undergo OAT. Group 2 consisted of ACI/NKyo rats that received OAT and were fed a normal chow diet and ROW beginning the day after surgery. Group3 consisted of ACI/NKyo rats that received OAT and were fed a normal chow diet and PAW beginning the day after surgery. Group 1 included 5 ACI/NKyo rats, sacrificed at day 150 after entering the experiment; group 2 included 15 ACI/NKyo rats, and 5 were randomly sacrificed at days 30, 60, and 90 after OAT; and group 3 included 20 ACI/NKyo rats, and 5 rats were randomly sacrificed at days 30, 60, 90, and 150 after OAT.

### 2.3. Orthotopic Aortic Transplantation

In brief, the donor PVG/Seac rats were anesthetized with xylaxin (2 mg/kg BW) mixed with Zoletil^®^ (containing a dissociative anesthetic, ketamine, and zolazepam at a ratio of 1:1.5 mg/kg BW). Continuous 100% oxygen mixed with 2% isoflurane was used to maintain anesthesia during the surgery. The fur was removed from the abdomen of the male PVG/Seac rats, the abdominal skin and muscles were carefully cut along the midline of the abdomen, and the organs in the abdomen were moved to one side. Then, the abdominal aorta was carefully located, the diaphragm was cut in a continuous extension, the thoracic aorta was located, and the tissues around the thoracic aorta, such as esophagus, nerves, and fat, were removed to avoid damaging the integrity of the thoracic aorta. Finally, approximately 1.5 cm of the free thoracic aorta was resected. The thoracic aorta was rinsed, and the blood was removed using normal saline. Then, the vessel was immersed in normal saline for a period not exceeding 20 min until use.

The male ACI/NKyo rats were anesthetized, the fur of the abdomen was removed, and the skin was sterilized. The abdominal skin and muscles were carefully cut along the abdominal midline, and the organs were moved to the outside of the body and wrapped with wet sterile gauze. Then, the abdominal aorta was located, the vessel was carefully freed, and the tissue and fat around the aorta were removed using a cotton swab. The small blood vessel branches were removed using a silk suture or coagulation device. It was necessary to maintain the integrity of the aorta and inferior vena cava. Then, 150 U/kg BW of heparin was injected into the tail vein, and the abdominal aorta was clamped to the lower part of the right renal artery and above the branch of the femoral artery using a bulldog vascular clamp. Approximately 1 cm (not exceeding 1.5 cm) of the aorta was cut using scissors. The donor and recipient aorta were anastomosed instead of anatomized with silk 7-O, after which the bulldog vascular clamps were released. The ischemia time was no more than 25 min. Finally, the abdominal organs were reset to their original positions and the abdominal muscles and skin were sutured using 6-O prolene. The complete OAT procedure was completed within 80 min. Appropriate analgesics and antibiotics were administered three days after the date of surgery.

### 2.4. Biochemical Measurements and Enzyme-Linked Immunosorbent Assays

Blood samples for biochemical measurements were collected from each animal prior to and at the end of the experiment. Samples were obtained from the tail vein, collected into tubes containing ethylenediaminetetraacetic acid or heparin, and plasma was separated by centrifugation at 1000× *g* for10 min at 4 °C. Plasma samples were stored at −80 °C until further use. Plasma total blood urea nitrogen (BUN), creatinine, alanine transaminase (ALT), aspartate aminotransferase (AST), blood sugar, and lactic dehydrogenase (LDH) were measured using a SPOTCHEMTM automatic dry chemistry system (SP-4410; Arkray, Shanghai, China). Enzyme-linked immunosorbent assays (ELISAs) were performed to determine the plasma levels of C-reactive protein (CRP; Abcam Inc., Cambridge, MA, USA), high mobility group box 1 (HMGB1; LifeSpan Biosciences, Inc., Seattle, WA, USA), transforming growth factor β1 (TGF-β1; Abcam Inc., Cambridge, MA, USA), SDF-1α (R&D Systems Inc., Minneapolis, MN, USA), interferon-γ (INF-γ; Abcam Inc., Cambridge, MA, USA), and interleukin-2 (IL-2; Abcam Inc., Cambridge, MA, USA).

### 2.5. Morphological Analysis

On the 30th, 60th, 90th, 120th, and 150th day of the experiment, the animals were anesthetized and sacrificed via cardiac injection of potassium chloride (100 mg/kg BW). The donor thoracic aorta and spleen transplanted into the recipient’s abdomen were then harvested, gently dissected free of adherent tissues, rinsed with ice-cold PBS, immersed and fixed in 4% buffered paraformaldehyde, paraffin embedded, and then cross sectioned for hematoxylin and eosin (H&E) staining and immunohistochemistry. The intimal and vessel wall area were calculated by ViewPoint software (Precipoint Inc., Thuringia, Germany). The ratio of intimal to vessel wall areas in the aortas from the five experimental groups was calculated. The thoracic aortas were also collected and stained with Picro sirius red for visualization of collagen accumulation. The slides were observed via polarized light microscopy. Additionally, immunohistochemical staining of thoracic aortas was performed using anti-αSMA (Santa Cruz Biotechnology, Dallas, TX, USA), anti-S100A4 (Cell Signaling Technologies, Danvers, MA, USA), anti-CD4 (Abcam Inc., Cambridge, MA, USA), anti-CD8 (Abcam Inc., Cambridge, MA, USA), and anti-CD11b (Abcam Inc., Cambridge, MA, USA) antibodies. Immunohistochemical staining of the spleen was performed using anti-CD4, anti-CD8, anti-CD11b, anti-CD20 (Abcam Inc., Cambridge, MA, USA), and anti-CD138 (Invitrogen, Thermo Fisher Scientific Co., Carlsbad, CA, USA) antibodies. All tissue slides used 5 mg/mL bovine serum albumin (BSA) for non-specific binding site masking for 1 h at room temperature. Sequentially, HRP-conjugated IgG was used to detect for 1 h after the specific antibody was used overnight at 4 °C, and 3,3′-diaminobenzidine (DAB)/H_2_O_2_-0.1 M Tris-HCl solution (0.5 mg/mL DAB and 0.01% H_2_O_2_) was used for color development. The slides were observed and the accumulation of positive stained cells was calculated in high power field (HPF, 400× magnification) using light microscopy.

### 2.6. Flow Cytometry

To investigate the effects of PAW on the mobilization of circulating EPCs and smooth muscle progenitor cells (SMPCs) in response to OAT, a fluorescence-activated cell-sorting flow cytometer (Becton Dickinson, San Jose, CA, USA) was used. A volume of 500 μL ACI/NKyo rat peripheral blood was incubated with Alexa Fluor 488-conjugated rabbit anti-rat CD133 (Novus Biologicals, Centennial, CO, USA), Cy5-conjugated rabbit anti-rat CD34 (Bioss Antibodies, Woburn, MA, USA), phycoerythrin (PE)-conjugated rabbit anti-rat alpha-smooth muscle actin (αSMA; Abcam Inc., Cambridge, MA, USA), and PE-conjugated mouse anti-rat vascular endothelial growth factor (VEGF; Novus Biologicals, Centennial, CO, USA) antibodies. Isotype-matched antibodies served as controls (Becton Dickinson, Franklin Lakes, NJ, USA). After incubation for 30 min, the cells were lysed (PharmLyse; Becton Dickinson, Franklin Lakes, NJ, USA), washed with phosphate-buffered saline (PBS), and fixed in 2% paraformaldehyde prior to analysis. Each analysis included 30,000 events. Circulating EPCs were considered to originate from the monocytic cell population and were gated by CD133^+^/CD34^+^/VEGF^+^ staining. In contrast, circulating SMPCs were gated byCD133^+^/αSMA^+^/CD34^−^ staining. Additionally, the percentage values of the circulating EPCs and SMPCs were represented as the ratio of monocytic cells.

### 2.7. Early EPC Colony-Forming Assay

Blood (2 mL) was obtained by heart puncture from euthanized animals. Total mononuclear cells (MNCs) were isolated by density gradient centrifugation using Histopaque-1083 (density: 1.083 g/mL; Sigma Aldrich, St. Louis, MO, USA). MNCs (5 × 10^5^ cells) were plated in 2 mL endothelial growth medium (EGM-2, Cat.: CC-3156; Clonetics Lonza, Alpharetta, GA, USA) on fibronectin-coated 6-well plates and cultured at 37 °C in a 5% CO_2_ incubator. After seven days of culture, the medium was changed, and non-adherent cells were removed; early EPCs remained attached to the plate. Thereafter, the medium was replaced every three days, and each colony/cluster was monitored through daily observation and calculation. A certain number of early EPCs from the control group continued to grow into mature EPC colonies.

### 2.8. Statistical Analyses

Values are expressed as the mean ± SD. Statistical evaluations were performed using Student’s *t*-test or one-way ANOVA, followed by Dunnett’s test. Results with a *p* value of <0.05 were considered statistically significant.

## 3. Results

### 3.1. Administration of PAW Did Not Affect the Biochemical Characteristics in OAT ACI/NKyo Rats

Biochemical analyses were performed to evaluate the effects of PAW on OAT ACI/NKyo rats. As shown in Table 1, body weight change, BUN, creatinine, ALT, and AST did not differ among the groups during the experimental period.

### 3.2. PAW Decreases Vasculopathy and Collagen Accumulation in OAT ACI/NKyo Rats

**Figure 1:**
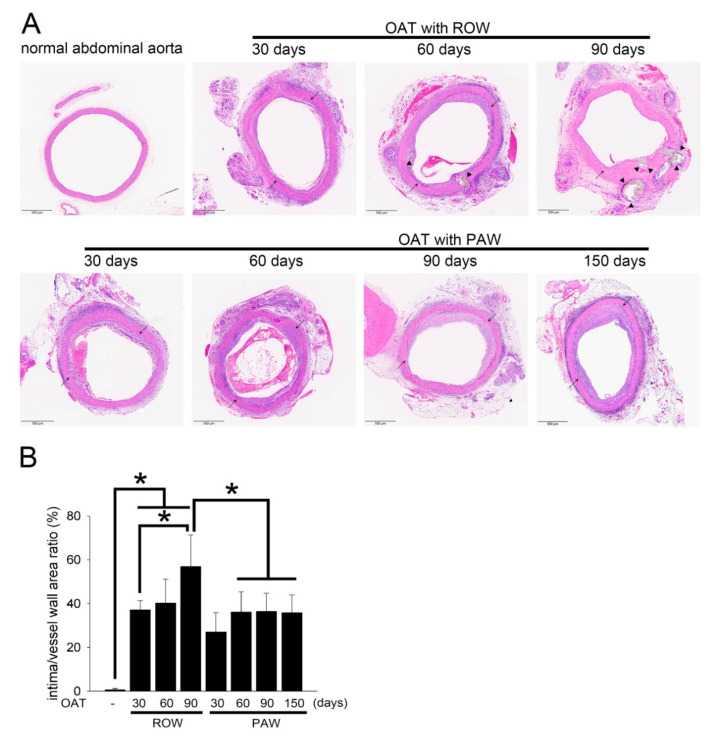

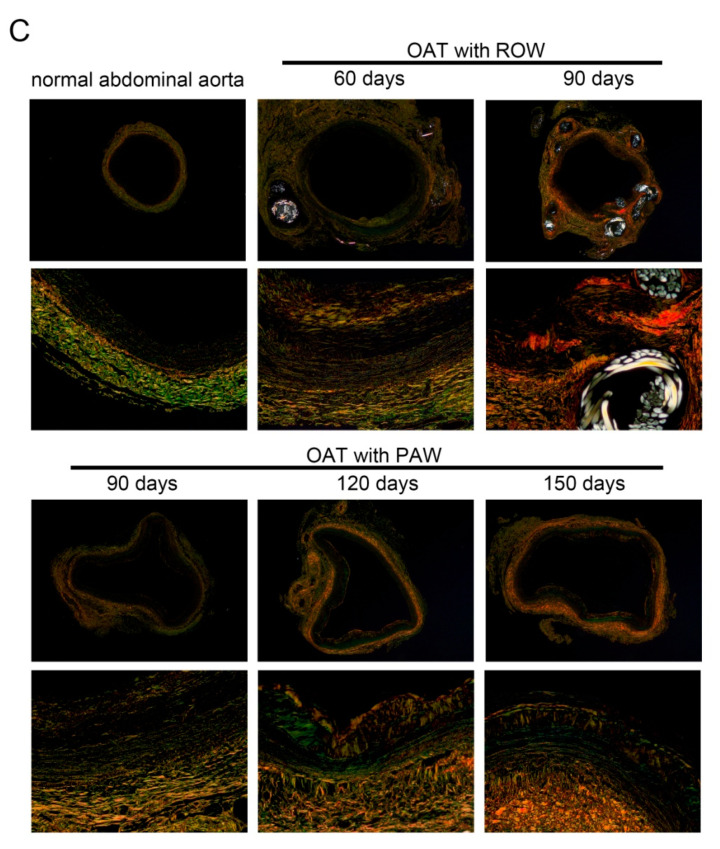
Administration of PAW in OAT ACI/NKyo rats resulted in reduced allograft vasculopathy. (**A**) Representative photographs of vasculopathy in thoracic aortas (from donor PVG/Seac rats) stained with H&E. The arrowheads indicate calcified lesions, and arrows indicate internal elastic lamina. (**B**) The graphs represent the ratio of intimal and vessel wall areas in the aortas from the five experimental groups. The images are 40× magnified. The results are expressed as the mean ± SD. Statistical evaluations were performed using the Student *t*-test, followed by Dunnett’s test. * *p* < 0.05 was considered as significant. (**C**) Histopathological features of thoracic aorta cross-sections were observed using Picro sirius red staining. The slides were observed via polarized light microscopy (40× (upper) and 200× (lower) magnification).

Representative photographs of vasculopathy in the thoracic aortas (from donor PVG/Seac rats) stained with H&E are shown in Figure 1A, and the quantifications of intima/vessel wall area ratio are shown in Figure 1B. No vasculopathy signs were observed in the aortas of normal PVG/Seac rats. Severe damage to vascular integrity, blurred elastin laminae, and accumulation of calcified plaques were markedly increased in the OAT group compared to normal vessels in PVG/Seac rats (Figure 1A). Additionally, the formation of an incomplete vascular structure correlated with time (from day 30 to day 90) in the OAT group. A significantly smaller area of aortic vasculopathy and greater vascular integrity were observed in the OAT plus PAW group compared to the OAT plus ROW group on day 90. Interestingly, the vessel wall remained intact, in addition to slight neointimal formation, at day 150 after OAT, and there was no calcified plaque formation.

Vasculopathy has been implicated in the development of collagen accumulation [19]. Therefore, Picro sirius red staining was performed for visualizing collagen. As shown in Figure 1C, normal thoracic aortas from PVG/Seac rats presented intact, thick collagen fibers (weak orange to red) and an equal distribution of fine collagen fibers (yellow to green) in the vessel walls. In contrast, the vessels in the OAT with ROW group at day 90 appeared to exhibit wall thickening in the inner layer, with some of the fine collagen fibers being piled up with a small amount of messy, thick collagen fibers. Moreover, the thick collagen fibers in the original vessel walls became inconsistent in thickness. However, in the OAT with PAW group, the disordered distribution and accumulation of fine and thick collagen fibers indeed improved significantly compared to that of the OAT with ROW group at day 90. The vessel wall showed reduced collagen accumulation which lasted up to day 120 and day 150 in the OAT plus PAW group.

### 3.3. Reduced Proliferation and Accumulation of Immune Cells Was Observed in the Vessel Wall of PAW Administered OAT ACI/NKyo Rats

**Figure 2:**
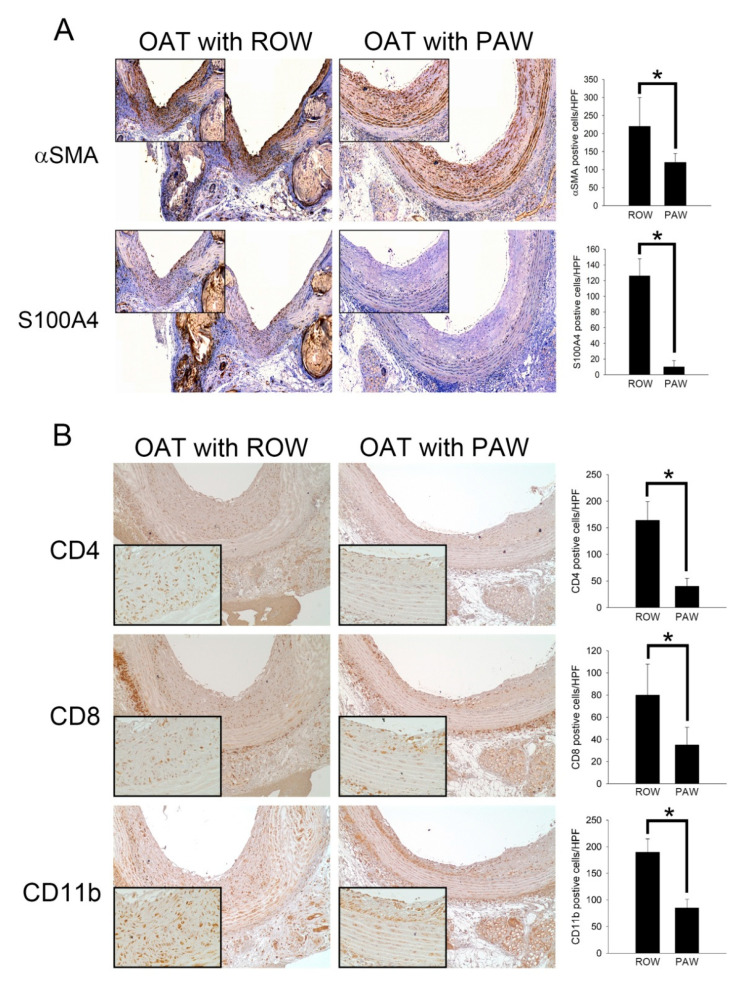
Administration of PAW is effective against SMCs, fibroblasts, CD4^+^ T lymphocytes, CD8^+^ T lymphocytes, and macrophage activity in OAT-induced chronic allograft vasculopathy. (**A**) Immunohistochemistry to assess proliferated SMCs (αSMA) and accumulated fibroblasts (S100A4) in rat thoracic aortas from donor PVG/Seac rats. The lumen is uppermost in all sections; the images are 200× magnified. Similar regions are shown as enlarged images (400× magnification) in the upper left corners. The brown signal indicates αSMA- and S100A4-positivecells. (**B**) Immunohistochemistry to analyze accumulated helper T cells (CD4), cytotoxic T cells (CD8), and infiltrated macrophages (CD11b) in rat thoracic aortas from donor PVG/Seac rats. The images are 200× magnified. Similar regions are shown as enlarged images (400× magnification) in the lower left corners. The brown signal indicates CD4-, CD8- and CD11b-positive cells. Semi-quantification of immunohistochemically positive stained cells in high power field (HPF) is shown in the right panel of (**A**,**B**). The graphs represent the accumulation of cells in the aortas of rats from the experimental groups. The results are expressed as the mean ± SD. Statistical evaluations were performed using Student *t*-test, followed by Dunnett’s test. * *p* < 0.05 was considered as significant.

Proliferation of SMCs and fibroblasts plays a critical role in the process of chronic allograft vasculopathy. Therefore, immunohistochemical staining was performed using antibodies against αSMA and S1000A4 on transplanted aorta sections (Figure 2A). The OAT PVG/Seac thoracic aorta sections at day 90 showed marked accumulation of SMCs and fibroblasts in hyperplasia areas on the luminal surface in the ROW-administered group compared to sections of the PAW-administered group. CD4- and CD8-positive T cells are subtypes of lymphocytes. CD4-positive T cells are MHC class II-restricted and CD8-positive T cells are a critical subpopulation of regulatory T-lymphocytes involved in MHC class I-restricted interactions, and hence they are important mediators of adaptive immunity. Infiltrated CD11b-positive macrophages also play critical roles in the process of chronic allograft vasculopathy. Therefore, immunohistochemical staining was performed on transplanted aorta sections to identify the immune reaction in the vessel using antibodies against CD4, CD8, and CD11b (Figure 2B). The thoracic aorta sections from the OAT PVG/Seac ROW-administered group at day 90 showed marked infiltration ofCD4-, CD8-, and CD11b-positive cells. However, T lymphocytes and macrophages were accumulated less in the vessel wall in PAW administered group. These results indicated that relatively minimal adaptive immune reactions occurred in the vessel wall of the PAW-administered group, resulting in reduced T lymphocyte and macrophage infiltration.

### 3.4. Administration of PAW Results in Lower Plasma Concentration of Inflammatory Factors and Cytokines in OAT ACI/NKyo Rats

As shown in Table 2, inflammation-related cytokines and markers were produced in the plasma. LDH is an enzyme that is released into the plasma following tissue injury, and might be strongly associated with antibody-mediated rejection [20]. Elevated plasma levels of LDH were observed in OATACI/NKyo rats administered with ROW; higher LDH levels (1888.78 ± 323.0 IU/L) compared to the baseline (854.6 ± 152.5 IU/L) were observed in the ROW group; the level of LDH was significantly higher than that of the non-OAT group (895.9 ± 125.6 IU/L) at day 90. However, the elevated LDH level was decreased in the OAT plus PAW group (968.8 ± 116.4IU/L) at day 90.CRP is an indicator of inflammation and tissue damage [21], and HMGB1 is involved in the chronic rejection of cardiac allograft vasculopathy [22]. As shown in Table 2, the CRP levels were increased after 90 days of OAT compared to the baseline in ROW (163.5 ± 22.5 mg/dL) or PAW (72.1 ± 5.4 mg/dL) plus OAT groups. Nevertheless, after OAT, PAW-administered rats still had relatively low levels of CRP compared to the rats administered with ROW. Similar to CRP levels, the HMGB1 expression was lower in rats in the PAW group than in the ROW group. Additionally, the function of EPCs is related to the occurrence of OAT-induced vasculopathy [23]. However, SDF-1α is involved in the homing and recruitment of EPCs, and TGF-β1 negatively regulates EPC function [24] following OAT. Regardless of whether the rats received ROW or PAW, OAT increased plasma SDF-1α and TGF-β1 levels. In particular, administration of PAW resulted in a significant SDF-1α increase (1211.4 ± 130.2 pg/mL) and TGF-β1 decrease (129.7 ± 54.1ng/mL) at day 90 compared to the OAT plus ROW group (432.6 ± 100.4 pg/mL for SDF-1α and 378.1 ± 55.9 ng/mL for TGF-β1). IFN-γ mediates transplant vasculopathy through CD8^+^ or CD4^+^T lymphocyte-associated injury of vascular endothelial cells. Furthermore, cytokines such as IL-2 cause a reversible insult to the endothelium around the time of transplantation [25]. Administration of PAW may significantly decrease IFN-γ (3.5 ± 1.2 pg/mL) and IL-2 (352.4 ± 95.7 pg/mL) production in ACI/NKyo rats after OAT for 90 days compared to the ROW-administered group. Moreover IFN-γ expression almost reaches the basal level in PAW-administered OAT ACI/NKyo rats. Based on these results, we predict that PAW may regulate alloimmunity and nonimmunity factors in an appropriate situation.

### 3.5. Administration of PAW Results in Reduced Cell-Mediated and Humoral Immune Responses inOAT ACI/NKyo Rats

**Figure 3:**
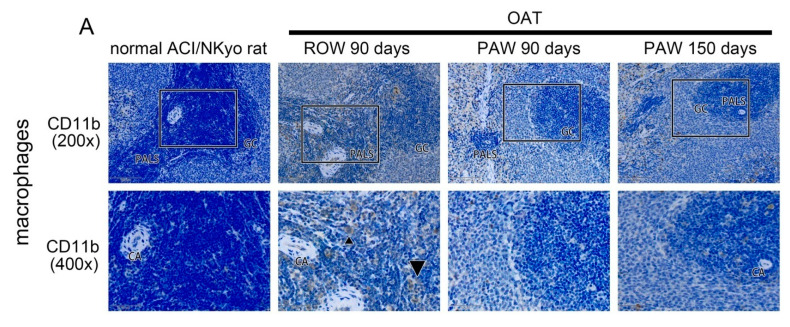

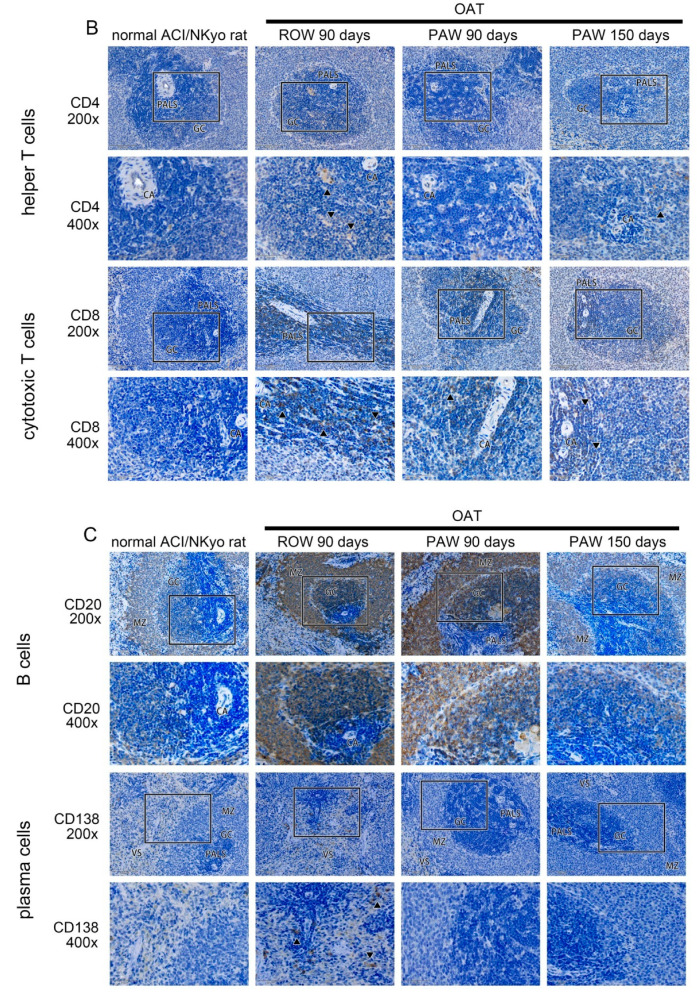
Decreased activity of macrophages, T lymphocytes, B lymphocytes, and plasma cells in the spleen of PAW administered OAT-ACI/NKyo rats. (**A**) Immunohistochemistry was performed to assess the accumulated macrophages (CD11b) in the spleen of OAT-recipient ACI/NKyo rats (GC, germinal center; PALS, periarterial lymphatic sheath; CA, central artery). The brown signals indicated by triangle arrow heads are CD11b-positive macrophages. The images in the upper column are 200x magnified, and the square regions are shown as enlarged images (400× magnification) in the lower column. (**B**) Immunohistochemistry was performed to analyze accumulated helper T cells (CD4) and cytotoxic T cells (CD8) in the spleen of OAT-recipient ACI/NKyo rats. The images are 200× and 400× magnified, respectively. The brown signal indicated by triangle arrow heads areCD4- and CD8-positivecells. (**C**) Immunohistochemical analysis of accumulated B cells (CD20) and activated plasma cells (CD138) in the spleen of OAT-recipient ACI/NKyo rats (MZ, mantle zone; VS, venous sinuses). The images are 200× and 400× magnified, respectively. The brown signals indicated by triangle arrow heads are CD138-positivecells. The cell nuclei were stained with hematoxylin, and the slides were observed via light microscopy.

Humoral and cell-mediated immunity are associated with the process of OAT-induced vasculopathy. Therefore, the spleens of experimental animals were analyzed by IHC to identify the severity of chronic rejection. CD11b-positive macrophage, which is also an antigen-presenting cell, activates the adaptive immune system [26]. As shown in Figure 3A, macrophages were barely observed in the germinal center (GC) and periarterial lymphatic sheath (PALS) of the spleen from naïve ACI/NKyo rats. In contrast, an increased number of macrophages appeared in the GC and PALS in the ROW-administered group after OAT for 90 days. However, after 90 and 150 days of OAT, PAW administration significantly reduced the accumulation of macrophages in the GC and PALS. There are two major subtypes of T lymphocytes, helper T (Th) cells (CD4^+^) and killer T (Tc) cells (CD8^+^), involved in cell-mediated immunity. As shown in Figure 3B, the GC and PALS of spleen of naïve ACI/NKyo rats did not accumulate CD4-positive Th cells. In addition, rats in the OAT plus ROW group presented increased accumulation of CD4-positive Th cells in the GC and PALS of their spleen, which decreased upon PAW administration. Similar to CD4-positive Th cells, CD8-positive Tc cells also infiltrated into the PALS of spleens in OAT plus ROW ACI/NKyo rats, which were reversed upon PAW administration. Additionally, B cells (CD20^+^) mediate humoral immune responses and differentiate into plasma cells (CD138^+^) to produce antibodies. As shown in Figure 3C, CD20-positive B cells predominantly cluster in the germinal center and mantle zone. Simultaneously, a reduced number of plasma cells were observed in the germinal center, mantle zone, and venous sinuses of the spleens of naïve ACI/NKyo rats. ACI/NKyo rats with OAT plus ROW administration presented a large number of CD20-positive B cells accumulated in the germinal center and mantle zone, and activated plasma cells were observed at venous sinuses. In ACI/NKyo rats with OAT plus PAW administration, longer duration of PAW administration resulted in decreased accumulation of CD20-positive B cells in the germinal center and mantle zone. However, plasma cells were not observed in the venous sinuses, germinal center, and mantle zone. Based on these results, we predict that administration of PAW might maintain low levels of cell-mediated and humoral immune responses in ACI/NKyo rats.

### 3.6. Administration of PAW Promotes Increased Mobilization of Circulating EPCs and Differentiation of Early EPCs Than Administration of ROW in ACI/NKyo Rats

**Figure 4:**
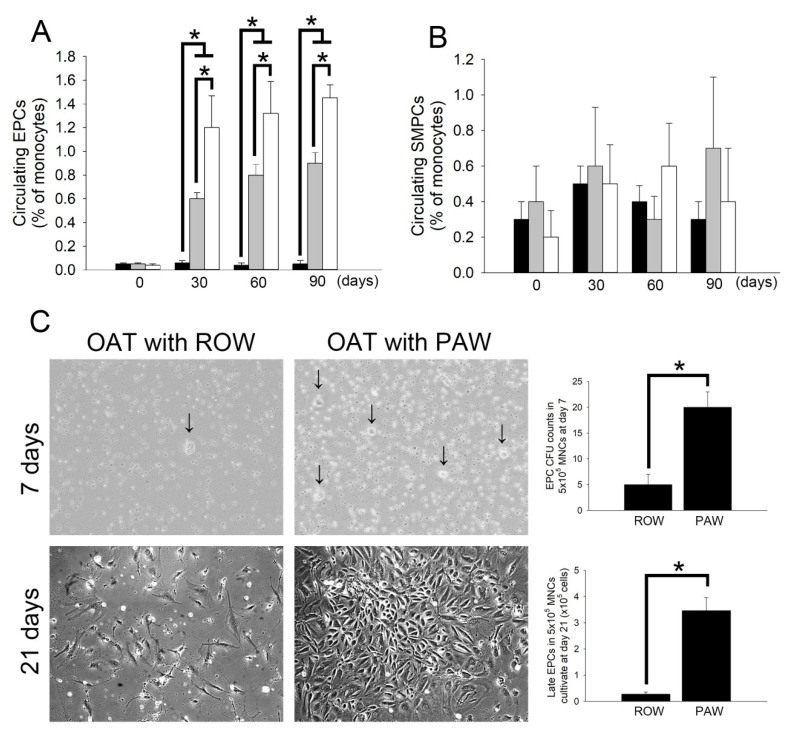
Administration of PAW promotes mobilization of circulating EPCs and differentiation of early EPCs in OAT-recipient ACI/NKyo rats. (**A**,**B**) EPC (defined as CD133^+^/CD34^+^/VEGF^+^ cells) and SMPC (defined as CD133^+^/αSMA^+^/CD34^−^ cells) mobilization at day 30, 60, and 90 following OAT in ACI/NKyo rats was determined by flow cytometry. Quantification of EPCs (left) and SMPCs in OAT-recipient rats (black bar, control group; light gray bar, OAT plus ROW administered group; white bar, OAT plus ROW administration group). (**C**) At the end of the experiment (90th day), MNCs from ACI/NKyo rats were seeded on 6-well plates. Cells were incubated with EGM-2 medium and observed every three days under a microscope beginning on day 7 after seeding the cells. Images at200× magnification on day 7 and day 21 of cultured cells are shown. Arrows indicate the total EPC colony-forming units (CFUs) over 7 days. Right graphs show the number of EPC CFUs (5 × 10^5^ MNCs/well in a 6-well plate) over 7 days, and late EPC numbers in 5 × 10^5^ MNCs cultured over 21 days in the different groups. All results are expressed as the mean ± SD (*n* = 5). Statistical evaluations were performed using Student’s test, followed by Dunnett’s test. * *p* < 0.05 was considered as significant.

EPCs play an important role in repairing damaged vessels during the process of OAT [27]. As shown in Table 2, administering rats with PAW after OAT resulted in increased SDF-1α and decreased TGF-1β as well as INF-γ production in ACI/NKyo rats. SDF-1α may mobilize circulating EPCs from the bone marrow [28]. TGF-1β is a guardian of T cell function [29] and may induce endothelial-mesenchymal transition (endo-MT) to ameliorate OAT-induced vasculopathy. INF-γ expression plays an important role in the progression of OAT-induced vasculopathy [30]. Therefore, following OAT surgery, the population of endogenous EPCs (defined as CD133^+^/CD34^+^/VEGF^+^ cells) and SMPCs (defined as CD133^+^/αSMA^+^/CD34^−^ cells) were quantified by flow cytometry, to compare the difference in levels of these cells between ROW- and PAW-administered ACI/NKyo rats. The results showed a significant increase in EPCs after 30 days of OAT in ROW-administered ACI/NKyo rats compared to that of non-OAT ACI/NKyo rats, and this increase was maintained until day 90 after OAT (Figure 4A). At day 30, OAT ACI/NKyo rats from the PAW-administered group exhibited a significantly increased number of mobilized EPCs in the peripheral blood compared to the OAT plus ROW group. However, the number of circulating EPCs was not associated with the time point after OAT in either ROW- or PAW-administered ACI/NKyo rats. Moreover, SMPCs initiated transplant arteriosclerosis. However, as shown by flow cytometry, the number of circulating SMPCs was not related to water administration, regardless of whether the animals received OAT (Figure 4B).

The capacity of MNCs to form EPC colonies may represent the differentiation ability of circulating EPCs. As shown in Figure 4C, the MNCs from ACI/NKyo rats seeded on fibronectin-coated wells on day 7 appeared to for mearly EPC colonies. MNCs from OAT ACI/NKyo rats administered with ROW and PAW also formed EPC colonies at 7 days of culture. The colonies of EPCs appearing on day 7 of culture presented as a central core of round cells with elongated sprouting cells at the periphery. However, rats receiving PAW exhibited more efficient differentiation of MNCs. After 21 days of continuous culture, EPCs had cobblestone-like morphology similar to that of mature endothelial cells and were confluent in the PAW-administered group. Conversely, the cells were sparse in the ROW-administered group (Figure 4C). These results indicated that PAW administration promotes increased mobilization and differentiation of early circulating EPCs more than administration of ROW in OAT-recipient ACI/NKyo rats.

## 4. Discussion

Water plays a vital role in all living entities and features as a solvent. In the human body, water functions as a medium for nutrient transfer and stabilizes body temperature levels. Additionally, water takes part in certain biochemical reactions of biochemical polymers, such as hydrolysis [31], glycogen disintegration, and adenosine triphosphate disintegration [32]. Water, a widely utilized solvent, is typically considered inert in chain reactions. Nevertheless, water, with its characteristic property of donor bridge acceptor for proton as well as electron transfer, has been considered as an appealing energetic reactant [33,34,35]. Surface plasmon resonance causes excitation of AuNPs to decay into energized hot electrons. Hot electron transfer can promote numerous chemical reactions [36], including dissociation of hydrogen [37], activation of oxygen [38], as well as electrocatalytic hydrogen evolution [39]. Our group proposed an innovative method to create PAW, which is prepared by treating ROW with resonantly excited AuNPs [1,40,41]. Recently, we have demonstrated that PAW is innovatively applicable in various fields [40]. PAW, with its unique properties of enhancing solute diffusion, as well as its exhibited anti-inflammatory properties, considerably shortens the time required to remove uremic contaminants during hemodialysis therapy [2,41]. Additionally, PAW decreased fibronectin expression and renal fibrosis in mice with chronic kidney disease [41], and reduced liver damage in chronic sleep-deprived rats [42]. Compared to mice administered with ROW, PAW administration may improve the survival time and cisplatin effect in Lewis-lung-carcinoma-cells-bearing mice, and may be associated with inducing Nrf2 gene expression [5]. A previous study suggests that PAW has potential therapeutic effects against Parkinson’s disease, periodontal disease, and Alzheimer’s disease [6].

After organ transplantation, once the immune and inflammatory responses are activated by alloimmune-, autoimmune-, or non-immune factors, the fibrotic changes that occur in the arteries of the graft result in OAT-induced vasculopathy, which subsequently causes ischemia [43]. Endothelium muscularization and intimal hyperplasia can be observed in most transplanted organs [44]. However, endo-MT refers to the process of differentiation of endothelial cells into interstitial cells (such as smooth muscle cells and fibroblasts) [45], which has been considered to be associated with the occurrence of OAT-induced vasculopathy [45,46,47]. A previous report has demonstrated that smooth muscle cell (SMCs) proliferation, mobilization and differentiation of circulating EPCs, and endo-MT coregulate the process of OAT-induced vasculopathy. The arteries of OAT-induced vasculopathy indicate the presence of SMCs, which are mostly derived from the donor [48] and are activated and proliferate following inflammatory responses. These cells exhibit chemotaxis moving from the media to the intima of the vessel [15]. In addition, donor-derived SMCs at the lesion originate from donor-derived ECs formed via endo-MT [49]. In recipients, bone-marrow-derived CD133^+^/CD34^+^/VEGF-R1^+^ EPCs and circulating CD31^+^/CD146^+^/vWF^+^/NOS^+^ EPCs have the characteristics of endothelial regenerative properties [50,51,52]. However, recent studies have found that recipients’ EPCs are, instead, involved in the pathological process of vasculopathy [11,53,54,55] due to the fact that circulating EPCs will adhere to the arterial endothelium and begin to proliferate, and induce persistent allogeneic immune responses [53]. In addition, EPCs will uncontrollably accumulate and induce massive proliferation of SMCs, and the proliferating SMCs will secrete large amounts of extracellular matrix proteins to cause intimal hyperplasia [51].Therefore, scientists speculate that despite the recipient exhibiting vigorousmobilization of EPCs, the persistent and uncontrolled allogeneic immune response may activate endo-MT and stimulate EPCs to differentiate into SMCs, which finally causes vasculopathy [53,54,55].The TGF-β signaling pathway is closely associated with the induction of fibrosis [45,56]. In our study, we found that OAT increases the plasma level of SDF-1α (Table 2) as well as the number of circulating EPCs in ACI/NKyo rats. However, PAW-administered ACI/NKyo rats had higher concentration of plasma SDF-1α and increased number of circulating EPCs compared to ROW-administered rats (Figure 4A). Although TGF-β1 is the major factor that induces endo-MT, the plasma TGF-β1 concentration in OAT-ACI/NKyo rats fed with PAW was lower than that of the ROW-fed group. Moreover, the decrease in circulating SMPCs in the PAW group was not statistically significant. We speculate that PAW may induce increased mobilization of EPCs from the bone marrow by increasing plasma SDF-1α concentration. On the other hand, PAW also reduces TGF-β1, includingTGF-β1-mediated endo-MT, which may be the possible mechanism of reducing OAT-induced vasculopathy. Endo-MT is regulated by complex molecular mechanisms. TGF-β1 and TGF-β2 promote endo-MT [55,57]. When endo-MT is induced by TGF-β, bone morphogenetic protein (BMP) and activin receptors, like kinase, regulate gene expression through phosphorylation of SMAD [58]. The phosphorylated SMAD enters the nucleus and interacts with key transcription factors that regulate endo-MT such as SNAI1, SNAI2, ZEB1, ZEB2, KLF4, TCF3, and TWIST, which ultimately leads to chromatin rearrangement and endo-MT-related gene expression [59]. In addition to TGF-β mainly activating SMAD to initiate the process of endo-MT, it may activate other complementary pathways including the mitogen-activated protein kinase pathway and phosphoinositide 3-kinase pathway, and inhibit the microRNA pathways that are related to endo-MT. Therefore, we further intend to analyze the molecular mechanisms involved in PAW-induced inhibition of endo-MT.

The DNA, RNAs, proteins, and so on in organisms are all biochemical polymers. In this study, we also analyzed the effect of PAW on the plasma levels of IFN-γ, HMGB1, and IL-2 in OAT-recipient rats. It is well known that IFN-γ expression is associated with activation, differentiation, and recruitment of T-cells via direct or indirect signaling pathways [58,60], which mediate OAT-induced vasculopathy. In 2016, Van Loosdregt and colleagues demonstrated that patients with transplanted heart vasculopathy had higher levels of IFN-γ, IFN-γ-inducible chemokines (fractalkine/CX3CL1, RANTES, ITAC, and IP-10), and IFN-γ-secreting Th1 cell markers (CXCR3, CCR5, and CX3CR1) in coronary arteries compared to control individuals [61]. Additionally, the nuclei of necrotic cells may release HMGB1 [62]; the mature antigen-presenting cells and macrophages are stimulated by IFN-γ or TNF-α to secrete HMGB1 [63,64]. In macrophages, the secretion of HMGB1 induces hyperacetylation of the protein [65] and increases the ability to participate in adaptive alloimmune rejection. HMGB1 may activate the differentiation of Th1 cells via receptors for advanced glycation end-products (RAGE) [63,66]. Stimulated endothelial cells in the vessel wall also produce HMGB1, and subsequently activate more endothelial cells and amplify the inflammatory responses [67,68]. Moreover, injury-induced mediators, such as IL-1 and C5a, may affect IFN-γ production and differentiation of Th1 cells. In general, the primary damage caused due to rejection may provoke a greater adaptive alloimmune response through specific mediators, thereby inducing T cells to produce increased IFN-γ and OAT-induced vasculopathy-related proteins, which ultimately leads to vasculopathy. In this study, we observed that administering PAW could almost reduce the plasma IFN-γ concentration in OAT-ACI/NKyo rats to similar levels to those of control ACI/NKyo rats. Although HMGB1expression did not decrease to the baseline level, it was significantly lower than the levels in ROW-administered OAT-ACI/NKyo rats. We speculate that PAW can effectively modulate the adaptive alloimmune response after OAT by modifying the expression of IFN-γ. However, since tissue damage and immune responses after OAT are caused by multiple immune factors, administering PAW can only partially reduce HMGB1 production in OAT-ACI/NKyo rats. The transferring plasmon effect does change physiological activities in rats. In the future, we consider using PAW in combination with existing clinical drugs to develop effective therapeutic strategies for OAT-induced vasculopathy.

In this study, although we only explored the effects of PAW on OAT-induced vasculopathy and obtained surprising results, we continue to study its detailed mechanism of action. Previous, we have demonstrated that dipeptidyl peptidase-4 (DPP-4) inhibitor lowers OAT vasculopathy in rats through direct increase in GLP-1 activity, and regulates SDF-1α levels and EPCs function [69]. We are the first group to provide new prevention and treatment strategies for DPP-4 inhibitors in chronic rejection-induced vasculopathy.

However, since drinking water is a natural activity that must be carried out in the lives of human beings, if drinking PAW only as a treatment method; most people will not be convinced. Therefore, without deviating from the current focus on prevention and treatment of OAT-induced vasculopathy, we are initiating a study to elucidate the synergistic effects of PAW on anti-rejection and immune modulator agents, such as DPP-4 inhibitor, and to analyze whether the use of PAW in combination with existing drugs can achieve better therapeutic effects than current treatment modalities. Overall, this study indicated that daily consumption of PAW might decrease the progress of vasculopathy in OAT-recipient ACI/NKyo rats by promoting the inhibition of collagen accumulation, SMC and fibroblast infiltration, and Tc and Th cell responses in the vessel walls. Furthermore, the data showed reduced activation of T lymphocytes, B lymphocytes, plasma cells, and macrophages in the spleens of PAW-administered OAT-recipient ACI/NKyo rats compared to ROW-administered OAT-recipient ACI/NKyo rats. Finally, we observed higher mobilization and levels of circulating EPCs in PAW-administered OAT-recipient ACI/NKyo rats compared to the control group.

## 5. Conclusions

**Figure 5:**
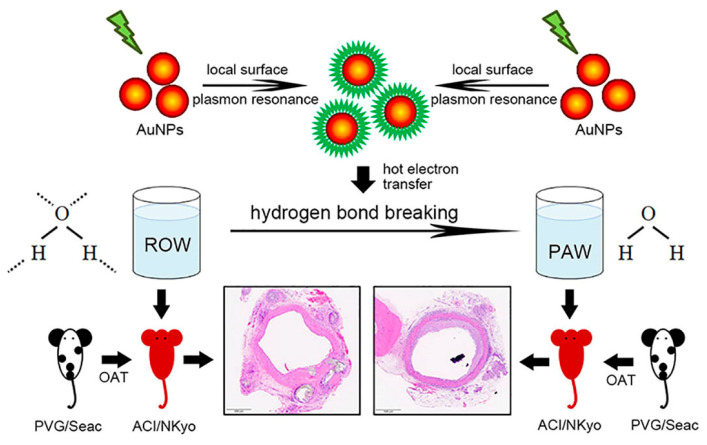
PAW may regulate chronic rejection-induced vasculopathy in OAT rats. Daily intake of PAW lowered the progression of vasculopathy in OAT-recipient ACI/NKyo rats by inhibiting collagen accumulation, proliferation of smooth muscle cells and fibroblasts, and T lymphocyte infiltration in the vessel wall. Moreover, the results showed reduced T and B lymphocytes, plasma cells, and macrophage activation in the spleens of the OAT-recipient ACI/NKyo rats that were administered PAW. Therefore, this study highlights the therapeutic roles of PAW and provides a more effective drug therapeutic route in vasculopathy.

As shown in Figure 5, PAW may regulate chronic rejection-induced vasculopathy in OAT rats. This study highlights the potential therapeutic roles of PAW in OAT-induced vasculopathy. Therefore, we look forward to more development, so that PAW can be applied to patients.

## Figures and Tables

**Table 1:** Comparison of Biochemical Parameters in Experimental ACI//NKyo Rats.

	Control ACI/NKyo (Non-OAT)		OAT + ROW (PVG/Seac to ACI/NKyo)			OAT + PAW (PVG/Seac to ACI/NKyo)		
	baseline	90 days	baseline	90 days	*p*	baseline	90 days	*p*
Body weight (g)	240.3 ± 18.6	350.1 ± 16.7	220.9 ± 12.8	332.5 ± 15.6	0.123	234.6 ± 10.2	365.7 ± 13.4	0.071
BUN (mg/dL)	26.5 ± 5.6	30.0 ± 5.9	26.8 ± 9.0	29.5 ± 6.1	0.898	25.4 ± 7.1	29.8 ± 6.8	0.956
Creatinine(mg/dL)	0.6 ± 0.1	0.7 ± 0.2	0.8 ± 0.5	0.8 ± 0.3	0.552	0.9 ± 0.3	0.9 ± 0.2	0.091
ALT (IU/L)	19.9 ± 6.7	22.8 ± 9.1	21.3 ± 6.5	23.5 ± 9.1	0.906	25.4 ± 3.8	20.6 ± 1.9	0.461
AST (IU/L)	30.5 ± 6.1	32.5 ± 8.1	30.5 ± 3.6	30.6 ± 8.1	0.720	29.8 ± 9.9	30.5 ± 9.1	0.685

OAT, orthotopic aortic transplantation; BUN, blood urea nitrogen; ALT, alanine transaminase; AST, aspartate transaminase. Values are represented as mean ± SD. *p* values are compared with Control ACI/NKyo (non-OAT) group at day 90. Statistical evaluations were performed using one-way ANOVA. *p* < 0.05 was considered statistically significant.

**Table 2:** Comparison of OAT-induced vasculopathy-related proteins in ACI//NKyo rats.

	Control ACI/NKyo (Non-OAT)		OAT + ROW (PVG/Seac to ACI/NKyo)		OAT + PAW (PVG/Seac to ACI/NKyo)	
	baseline	90 days	baseline	90 days	baseline	90 days
LDH (IU/L)	900.5 ± 99.5	895.9 ± 125.6	854.6 ± 152.5	1888.78 ± 323.0 ^a,b^	802.3 ± 152.0	968.8 ± 116.4 ^c^
CRP (mg/dL)	40.2 ± 13.5	39.6 ± 9.1	39.4 ± 9.8	163.5 ± 22.5 ^a,b^	26.5 ± 8.1	72.1 ± 5.4 ^a,b,c^
HMGB1 (ng/mL)	2.7 ± 1.4	2.8 ± 1.3	3.4 ± 1.5	98.7 ± 19.7 ^a,b^	2.5 ± 1.4	55.4 ± 13.1 ^a,b,c^
SDF-1α (pg/mL)	200.3 ± 22.6	196.5 ± 52.1	220.1 ± 41.5	432.6 ± 100.4 ^a,b^	241.3 ± 54.1	1211.4 ± 130.2 ^a,b,c^
TGF-β1 (ng/mL)	66.3 ± 21.2	78.9 ± 25.4	72.4 ± 19.7	378.1 ± 55.9 ^a,b^	72.1 ± 22.0	129.7 ± 54.1 ^a,b,c^
INF-γ (pg/mL)	2.5 ± 0.8	3.1 ± 0.9	1.5 ± 0.7	18.7 ± 5.3 ^a,b^	1.9 ± 0.8	3.5 ± 1.2 ^c^
IL-2 (pg/mL)	45.7 ± 13.5	56.8 ± 18.9	53.7 ± 21.5	899.4 ± 102.4 ^a,b^	45.2 ± 21.0	352.4 ± 95.7 ^a,b,c^

OAT, orthotopic aortic transplantation; LDH, lactic dehydrogenase; CRP, C-reactive protein; HMGB1, high mobility group box 1 protein; INF-**γ**, interferon **γ**; TGF-β1, transforming growth factor-beta 1; SDF-1α, stromal cell-derived factor 1α; IL-2, interleukin 2. Values are represented as mean ± SD. ^a^ *p* < 0.05 compared with baseline of the same group; ^b^ *p* < 0.05 compared with control ACI/NKyo (non-OAT) group at the same time point; ^c^ *p* < 0.05 compared with OAT + ROW (PVG/Seac to ACI/NKyo) group at the same time point. Statistical evaluations were performed using one-way ANOVA.

## Data Availability

The data presented in this study are available on request from the corresponding author.
